# Semiconductor-Based Photoelectrocatalysts in Water Splitting: From the Basics to Mechanistic Insights—A Brief Review

**DOI:** 10.3390/ma18091952

**Published:** 2025-04-25

**Authors:** W. J. Pech-Rodríguez, Nihat Ege Şahin, G. G. Suarez-Velázquez, P. C. Meléndez-González

**Affiliations:** 1 Department of Mechatronics, Polytechnic University of Victoria, Ciudad Victoria 87138, Tamaulipas, Mexico; 2 Battery and Materials, Department of Biological and Chemical Engineering, Aarhus University, 8200 Aarhus, Denmark; 3 Departamento de Ingeniería en Energía, Universidad Politécnica de Altamira, Nuevo Libramiento Altamira Km. 3, Santa Amalia, Altamira 89602, Tamaulipas, Mexico; gladis.suarez@upalt.edu.mx; 4 Tecnológico Nacional de México, Instituto Tecnológico Del Valle de Etla, Abasolo S/N, Barrio Del Agua Buena, Santiago Suchilquitongo, Oaxaca 68230, Oaxaca, Mexico; 5 Department Interface Design, Helmholtz-Zentrum Berlin für Materialien und Energie GmbH (HZB), Albert-Einstein-Straße 15, 12489 Berlin, Germany

**Keywords:** photoelectrocatalyst, water oxidation, semiconductors, NiWO_4_, Fe_2_O_3_, water splitting

## Abstract

Hydrogen and oxygen serve as energy carriers that can ease the transition of energy due to their high energy densities. Nonetheless, their production processes entail the development of efficient and low-cost storage and conversion technologies. In this regard, photoelectrocatalysts are materials based on the photoelectronic effect where electrons and holes interact with H_2_O, producing H_2_ and O_2,_ and in some cases, this is achieved with acceptable efficiency. Although there are several reviews on this topic, most of them focus on traditional semiconductors, such as TiO_2_ and ZnO, neglecting others, such as those based on non-noble metals and organic ones. Herein, semiconductors like CdSe, NiWO_4_, Fe_2_O_3_, and others have been investigated and compared in terms of photocurrent density, band gap, and charge transfer resistance. In addition, this brief review aims to discuss the mechanisms of overall water-splitting reactions from a photonic point of view and subsequently discusses the engineering of material synthesis. Advanced composites are also addressed, such as WO_3_/BiVO_4_/Cu_2_O and CN-FeNiOOH-CoOOH, which demonstrate high efficiency by delivering photocurrent densities of 5 mAcm^−2^ and 3.5 mA cm^−2^ at 1.23 vs. RHE, respectively. Finally, the authors offer their perspectives and list the main challenges based on their experience in developing semiconductor-based materials applied in several fields. In this manner, this brief review provides the main advances in these topics, used as references for new directions in designing active materials for photoelectrocatalytic water splitting.

## 1. Introduction

Due to the undeniable increase in global energy demand, projected to reach more than 51,000 TWh by 2050, society is relying on the use of alternative technologies, such as solar cells, wind turbines, photoelectrochemical (PEC) cells, and others [1,2]. Among these, PEC cell technology is gaining significant interest due to its environmental friendliness, along with its sustainability and capability in generating hydrogen, as chemical energy, through the water-splitting process [3,4,5]. In principle, the PEC water-splitting process combines photochemistry and electrochemistry, namely photoelectrocatalysis, which relies on producing sustainable hydrogen by employing sunlight and water. This process involves capturing photoelectrons and generating charge carriers that drive the two-half reactions of water splitting: (i) the water oxidation reaction occurring at the photoanode, and (ii) the reduction of the photons taking place at the photocathode [6,7]. Owing to the reaction sites being spatially separated, these cells reduce the process costs and technical challenges. They hold promise when solar energy is utilized, given its abundance and ready availability in nature [8,9]. The main barrier for traditional photocatalysts is their low effectiveness and stability during the water-splitting process [10,11,12]. In fact, a recent work published by Kuchipudi et al. [13] stated that their poor efficiency is mainly due to the wide band gap that is in the UV region, as this only accounts an 5% of the solar spectrum. On the other hand, it is well known that the poor kinetics of the oxygen evolution reaction lead to the low efficiency of PEC water splitting. This is partly why many materials have been tested, and scavenger molecules are often used to address this issue [14]. For example, notable reviews have been published exploring innovations in the photoelectrocatalytic field, including the use of biomolecules to enhance hydrogen production efficiency [15,16,17]. In fact, PEC cells are gaining attention, as they can mitigate electron–hole pair recombination by applying a small external bias potential, thereby enhancing their overall performance [18]. Additionally, electrocatalysts, such as metal oxides and metal–organic frameworks, have been incorporated into the matrix of semiconductive materials to obtain photoelectrocatalysts or composites that can boost the rate of hydrogen production due to simultaneous mechanics occurring [19,20,21].

By directly harnessing solar energy and integrating light absorption with photoelectrocatalysts in a single device, the PEC system has become an alternative to generating sustainable H_2_ and O_2_ at a large scale with low costs. Thus, from the techno-economic point of view, the utilization of PEC cells is more competitive with fossil-based primary technologies. Furthermore, it is important to note that H_2_ and O_2_ production in PECs can occur simultaneously with other reactions of interest, such as the degradation of harmful molecules [22,23,24]. Numerous reviews in the literature highlight the significance of developed materials and their efficiency in terms of overpotential and photocurrent. However, few of them correlate these parameters or provide an in-depth discussion of the underlying mechanisms [1,25,26]. This review provides an in-depth discussion of the fundamentals of PEC cells, with a focus on semiconductor mechanisms. Subsequently, an analysis of recent findings is presented, emphasizing their optoelectronic, morphological, and photoelectrochemical properties. The effectiveness of various photoelectrocatalysts, such as CdSe, NiWO_4_, and Fe_2_O_3_, is also compared to identify drawbacks and explore potential improvements. For example, charge transfer resistance (R_ct_) in photoelectrocatalysts is one of the key factors considered, and its values are thoroughly examined across different materials. In addition, design and engineering material, operational conditions, and controlling surface modification are considered during the discussion of each subsection. Finally, the main barriers to this technology, along with future perspectives on this technology, are addressed, based on the experience of the authors.

## 2. Fundamentals of PEC Technology and Characterization

### 2.1. Fundamentals of PEC Water Splitting

**Figure 1:**
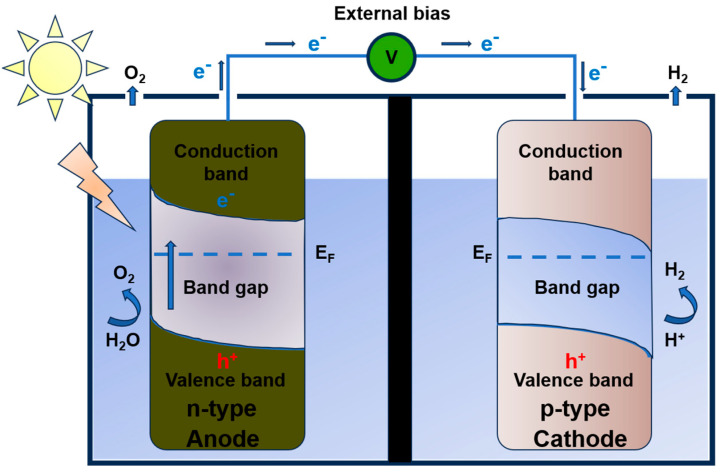
A schematic representation of the basic operational principles of photoelectrochemical water oxidation in a PEC cell with two semiconductor electrodes, reproduced under the terms and conditions of the Creative Commons Attribution (CC BY) license (https://creativecommons.org/licenses/by/4.0/, accessed on 13 January 2025) [34].

A PEC cell is a device consisting of a semiconductor photoanode that absorbs sunlight and generates electron–hole pairs, a metal or metal oxide photocathode, and an electrolyte. These components are interconnected by an external circuit, facilitating the electron transfer, as shown in Figure 1. The anode typically contains an *n*-type semiconductor, which produces a positive photocurrent, whereas the cathode may be composed of a *p*-type material that delivers a negative photocurrent [27,28]. It is important to note that in non-exited semiconductors, the valence band is nearly full, while the conduction band remains almost empty [29,30]. However, when external energy is applied (such as an increase in temperature, light irradiation, or a bias voltage), electrons can gain sufficient energy to transition from the valence band to the conduction band, leaving behind unoccupied states in the valence band called holes [31]. When the PEC cell device is illuminated with a wavelength that matches the semiconductor bandgap, an electron–hole pair is produced within the material, along with an internal electric field [32]. The electrons are transferred through the external circuit to the photocathode, where hydrogen is produced, while the holes migrate to the semiconductor–electrolyte interface, driving the oxygen evolution reaction [33].

As is well known, water splitting requires at least 1.23 eV vs. SHE (at 25 °C and 1 atm), which is the required value for reaching the O_2_ evolution, so the implemented semiconductor in a PEC cell must be capable of managing photons with energy greater than this voltage [35]. For practical application, heat generated by the reaction increases the required potential to approximately 1.48 eV. The difference between these two potentials is commonly referred to as the overpotential [36]. During the overall water-splitting process (Equation (3)), two half-reactions occur in parallel at the anode and the cathode. Equations (1) and (2) describe, respectively, the oxygen evolution reaction (OER) at the anode and the hydrogen evolution reaction (HER) at the cathode in an acidic medium [10,37].

Anode:

(1) $$2H_{2}O\left(l\right)\to O_{2}\left(g\right)+4H^{+}+4e^{-}$$

Cathode:

(2) $$4H^{+}+4e^{-}\to 2H_{2}(g)$$

Overall reaction:

(3) $$2H_{2}O\left(l\right)\to O_{2}\left(g\right)+H_{2}(g)$$

It should be considered that not all photons are absorbed by the semiconductor and this excess of energy is transformed, mainly as heat and light, thus affecting the overall cell efficiency. Another crucial parameter to consider is the conductivity of the photoelectrocatalysts and their ability to prevent rapid electron–hole recombination. PEC efficiency can be evaluated from different perspectives, with solar-to-hydrogen (STH) conversion being one of the most commonly used methods to assess the material’s effectiveness. It is calculated using Equation (4) [38,39,40].

(4) $$STH=\frac{mol\;H_{2}\left(m^{-2}s^{-1}\right)\times 237\;kJ\;mol^{-1}}{P_{in}(W\;m^{-2})}=\frac{j_{sc}\left(Am^{-2}\right)*1.23V\times \eta_{F}}{P_{in}(W\;m^{-2})}$$

where *j_sc_* is the short circuit current, *P_in_* is the incident illumination (100 mW m^−2^), and *η_F_* is the faradaic efficiency. Another method for estimating the efficiency of PEC cells is by determining the incident photocurrent efficiency (IPCE), which describes the photocurrent dependence as a function of wavelength. It is calculated using the following equation [41,42,43]:

(5) $$IPCE(\lambda )=\frac{j_{ph}\left(Am^{-2}\right)\times 1239.8(V\;nm)\;}{P_{monoAM1.5G}\left(W\;m^{-2}\right)\times \lambda \;(nm)}$$

where *j_ph_* is the photocurrent density, *P_mono_* is the monochromatic illumination, and *λ* is the source wavelength. The determination of PEC cell efficiency is crucial for the design and fabrication of novel photoelectrocatalysts, as it serves as the key performance metric when optimizing parameters such as defects, vacancies, band gap, flat band potential, and other material properties.

### 2.2. Electrochemical Characterization

Various electrochemical techniques have been proposed to determine the photoelectroactivity of photoanodes, as well as to assess the stability of the materials during the water-splitting process [44]. It is important to mention that the test conditions, like electrolyte type, temperature, light source, and others, depend on the intended application of the photoelectrode. In general, alkaline electrolytes, such as KOH, NaOH, and Na_2_SO_4_, are commonly used because they enhance conductivity at a pH of 14 and provide OH^−^ species that interact with active sites, improving water oxidation [45]. These electrochemical tests are typically performed using a potentiostat with a three-electrode setup. The working electrode can be a modified vitreous carbon electrode, where the photoelectrocatalyst is deposited, or an FTO glass substrate containing the material. Reference electrodes, such as Ag/AgCl, Hg/HgO, or RHE, are used to monitor and facilitate the sensing of reactions occurring within the electrochemical cell. Meanwhile, the counter electrode might be a Pt wire or graphite bar. Measurements are typically conducted under illumination conditions, using solar simulators or visible–UV lamps with intensities approaching 100 mW cm^−2^, simulating the standard AM 1.5 solar spectrum.

**Figure 2:**
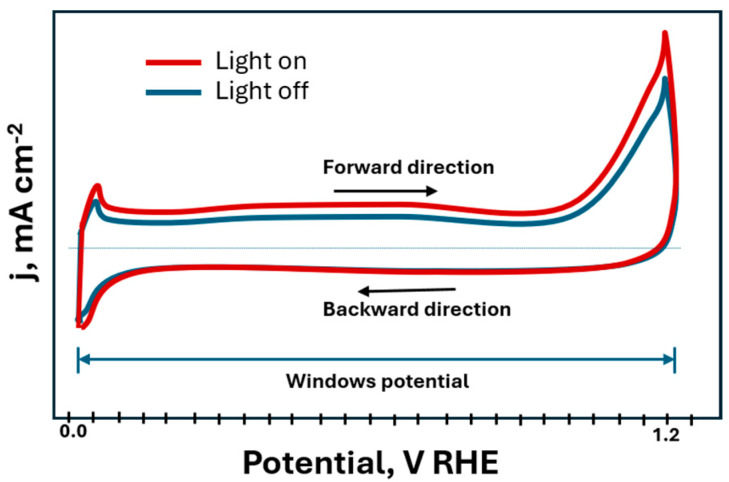
Scheme of representation of CV plot for photoelectrocatalysts.

Cyclic Voltammetry (CV) is a technique used to evaluate the electrocatalytic activity and redox processes in photoelectrodes. It also determines the window stability of the material under illumination and enables the study of charge transfer kinetics and the reversibility of surface reactions [46]. Figure 2 illustrates CV plots where the *x*-axis represents the explored potential windows of the test, while the *y*-axis is the obtained current density attained by the material. If the material is photoactive, the CV plot may show changes in both the potential value and current densities.

**Figure 3:**
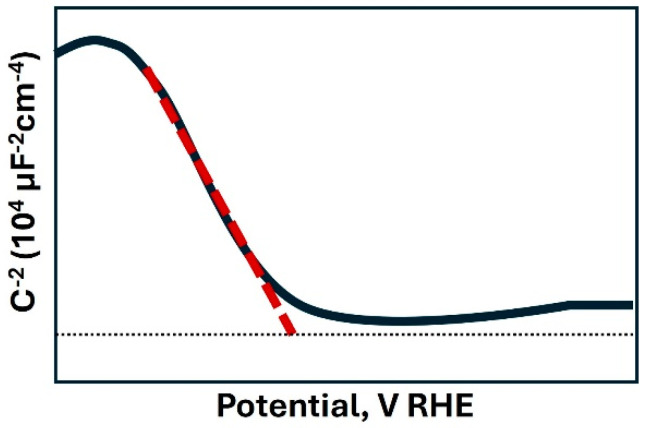
Scheme of Mott–Schottky plot.

Low-cost semiconducting materials, including TiO_2_, Fe_2_O_3_, BiVO_4,_ and Cu_2_O, among others, are usually developed in the form of thin films. For example, Thach Khac Bui et al. [47] fabricated g-C_3_N_4_-decorated TiO_2_ nanotubes, which demonstrated favorable performance in the hydrogen evolution reaction, with an overpotential of 0.9 V at a current density of 10 mA cm^−2^. Using the CV technique, they performed multiple scans to analyze the relationship between capacitance and active surface area. Quan Li et al. [48] developed BiVO_4_ photoanodes decorated with Ti_3_C quantum dots and used CV to determine the electrochemically active area (EASA) in the non-faradic region, a key parameter in evaluating the catalytic activity of the electrocatalyst, in addition to complementing the analysis with other techniques. On the other hand, Mott–Schottky (MS) analysis is a well-established electrochemical technique used to evaluate capacitance as a function of potential (Figure 3) and is mainly used for the calculation of charge carrier density (N_d_) in semiconductor materials. Additionally, this method enables the determination of the flat band potential, bandgap energy, and Fermi level [49]. From this measurement, it is possible to confirm the type of semiconductor to which the photoelectrode belongs, either *n*-type for a positive slope or *p*-type for a negative slope [50]. To obtain these curves, frequencies depending on the photoelectrode are applied, generally in a range between 1 kHz and 100 kHz [51]. In Figure 3, the Mott–Schottky plot exhibits the typical behavior of a *p*-type semiconductor characterized by a decrease in the reciprocal of the square of the capacitance as the applied potential increases, as a result of a reduction in the depletion region.

**Figure 4:**
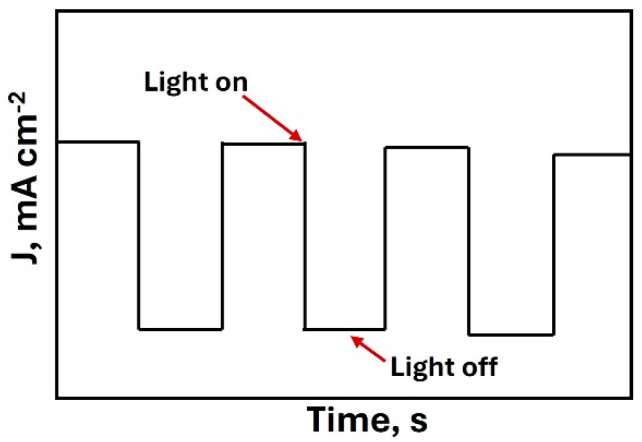
Scheme of CA with cycles light on and light off.

In this regard, Sarita Mittal et al. [52] developed a SnS_2_/g-C_2_N_2_N_4_ heterojunction to improve photoelectrochemical water separation. MS plots revealed a positive slope, indicating the characteristic behavior of an *n*-type semiconductor and confirming that the slope is proportional to the carrier density. In addition, they determined the flat band potential by extrapolating the curve to the point where the capacitance equals zero. On the other hand, Karolina Syrek et al. [53] used WO_3_-SeO_2_-CuO anodic photoelectrodes, operating them under solar illumination. Using MS analysis at 1 kHz, they confirmed that the materials exhibited *n*-type behavior due to their positive slopes. In addition, they observed shifts in the curves when comparing materials synthesized with different selenium concentrations. These shifts were attributed to the flow of electrons from the conduction band of SeO_2_ toward WO_3_, which contributes to the inhibition of electron–vacancy pair recombination. Therefore, it can be inferred that the MS technique is a valuable tool for characterizing the semiconductive behavior of materials, as it allows for the determination of not only the conductivity type but also the flat-band potential and capacitance. Another useful technique is chronoamperometry (CA), which is performed at a constant potential while measuring current as a function of time. This method allows for the evaluation of the photoelectrochemical stability of photoelectrodes under continuous operation and the analysis of material degradation and stability. Besides, it is used to study the transient photoresponse of the material with light–dark cycles, by switching the illumination on and off, which provides fast evidence of photoactivity [54]. As shown in Figure 4, in general, measurement times are between 10 min to 10 h or more. Gopichand Talasila et al. [55] developed photoelectrodes with metal oxide heterostructures, including BiVO_4_, Fe_2_O_3_, Co_3_O_4,_ and WO_3_. Using the CA technique, in 0.1 mol L^−1^ NaOH under chopped light and dark illumination, they analyzed the response of these heterostructures. BiVO_4_ achieved a high photocurrent density of 1.68 mA cm^−2^, while Fe_2_O_3_/Co_3_O_3_O_4_ exhibited a dark current density of 4.95 mA cm^−2^.

## 3. Photoelectrocatalysis

### 3.1. Traditional Semiconductive Photoelectrocatalysts

**Figure 5:**
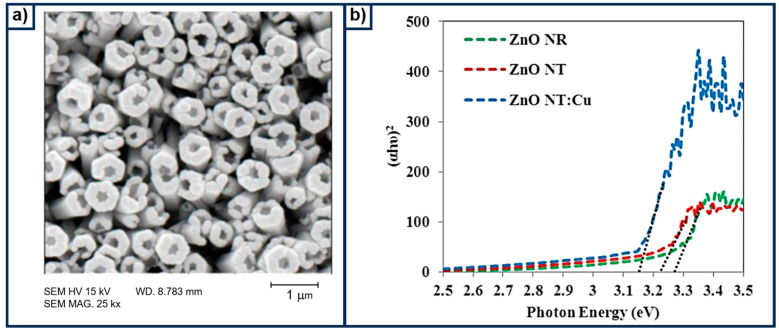
Characterization of the Cu-doped ZnO nanotubes. (**a**) An FESEM image of the electrodeposited Cu on the ZnO nanotubes, and (**b**) the band gap determination from the Tauc plot for the pristine and modified sample. Reproduced under the terms of ANALYTICAL & BIOANALYTICAL ELECTROCHEMISTRY (http://www.abechem.com, accessed on 14 January 2025). Copyright © 2024 by CEE (Center of Excellence in Electrochemistry) [63].

The implementation of semiconductive materials to conduct water splitting was reported some years ago [56,57,58,59]. For example, TiO_2_ is one of the most commonly studied compounds, since its first studies that were conducted by Fujishima and Honda [60,61], considering that it is easy to obtain and produce photoactivity that can be measured by traditional instruments. Nonetheless, due to the inappropriate band gap and electronic features of primary semiconductors, some surface engineering or chemical modification has been introduced. From that point on, several attempts have been made to improve the efficiency of these materials in water oxidation, with the addition of layers containing other metals or organic compounds being one approach. Yang et al. [62] reported on the alteration of TiO_2_ nanowire arrays by adding a CoO-Co_3_O_4_ layer. Photoelectrochemical characterization showed that the modified photoanode was capable of reaching a current density of 0.23 mA cm^−2^ at 1.23 V, which was superior to the pristine TiO_2_ sample, at 0.17 mA cm^−2^. The determined charge transfer resistance (R_ct_) was 5375 Ω and 8359 Ω for the modified and the raw TiO_2_, respectively. Although the author does not mention the calculated band gap from the UV–vis absorption spectra, it can be inferred that both TiO_2_ and the modified one with CoO-Co_3_O_4_ modified TiO_2_ have a band gap above 3 eV. Therefore, this slight enhancement is attributed to the ability of the cobalt oxide to improve visible light absorption, as was observed in the spectra. A similar attempt was adopted by Mollaei [63], who added an electrodeposited film of copper on ZnO nanotubes. This process was quite simple but successful in obtaining an active photoanode. Figure 5a depicts the Field Emission Scanning Electron Microscopy (FESEM) image for the ZnO nanotubes modified with Cu, where it is possible to observe the voids of each nanotube that were vertically aligned. This morphology is important, as water splitting in photoelectrochemical cells relies on surface–electrolyte interactions. Therefore, porous structures can enhance the process. Moreover, incorporating Cu into ZnO not only alters its chemical and structural characteristics but also affects its optical properties, as suggested by the observed bandgap modification, as shown in Figure 5b. The determined band gap for the ZnO modified with Cu was 3.15 eV, which is still large, limiting the ability of the material to effectively absorb visible light from the sunlight. Thus, adding Co into TiO_2_ or Cu into ZnO results in a slight photocatalytic improvement mainly due to the band gap modulation and electronic tuning. This approach was among the first strategies aimed at developing more efficient photoelectrocatalysts.

Araujo and coworkers [64] studied the effect of substituting 10% Cu with Ag species in the multielement Cu_2_ZnSn(S, Se)_4_ composite. The obtained photocathode not only reduced the Cu-related defects but also revealed a positive shift in the onset potential, at 0.2 V vs. RHE, that resulted in an enhancement of the photocurrent density by three times, with an STH efficiency six times higher than the unmodified one. The authors stated that the improvement was due to a morphological effect, where the grain expansion along the depth direction was influenced by the larger size of the Ag cation. This information was rapidly corroborated by the magnified XRD diffractogram, focusing on a range from 26.8° to 27.6° 2theta, which clearly showed the shifted peak position of the (112) plane. Surprisingly, the material exhibited a very small band gap of 1.11 eV, demonstrating the effectiveness of the doping approach in developing highly active materials. As deduced from the presented information, using TiO_2_, ZnO, or SnO_2_ alone as a photoelectrocatalyst in PEC cells results in lower efficiency, making real-world applications challenging. This is mainly attributed to their wide band gap, combined with a high R_ct_, a common issue observed in semiconductors, such as TiO_2_, ZnO, and SnO_2_. Therefore, significant efforts have been made to explore viable alternatives, such as doping, surface treatment, and the creation of heterojunctions, to enhance efficiency and overcome the limitations of large band gaps that primarily absorb UV light. These aspects are discussed below, highlighting relevant studies that thoroughly examine the synthesis methods, efficiency, and mechanisms involved in the water-oxidation process.

### 3.2. CdSe-Based Photoelectrocatalysts

**Figure 6:**
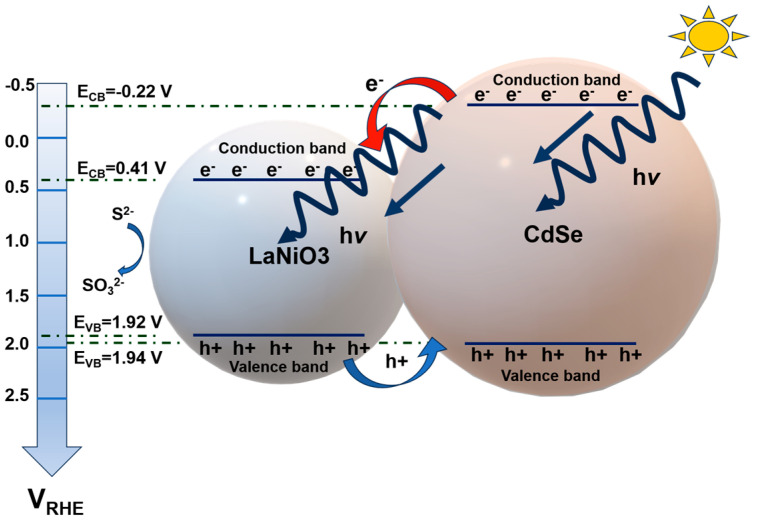
Schematic diagram of energy band mechanism presented in LNO@CdSe photoanode. Copyright 2023 Elsevier, reproduced with permission from reference [13].

Cadmium selenide (CdSe) is attracting significant interest due to its uniform particle size distribution, which can be activated across a wide range of wavelengths, mainly within the visible spectrum region. It is also classified as an II-VI semiconductor due to its direct band gap of 1.74 eV at 300 K (enough energy to split water) and can exist in both cubic and wurtzite crystal structures [65,66]. This material has been proposed for several applications, including sensors, solar cells, and PEC cells [67,68]. An interesting work reported the development of a highly active heterostructure composed of LaNiO_3_ (LNO) sensitized with CdSe quantum dots [13]. The composite was developed in two consecutive steps, where LaNiO_3_ was prepared by adopting the sol–gel method and using La (NO_3_)_3_⋅6H_2_O, Ni (NO_3_)_2_⋅6H_2_O as chemical precursors, in the presence of citric acid and NH_4_OH. Thereafter, CdSe QDs were synthesized by the hot-injection method. Finally, the composite was fabricated by 3-mercaptopropionic acid (3-MPA, with the chemical formula C_3_H_6_O_2_S). Figure 6 depicts the mechanism proposed by this research group for photoelectrochemical water splitting using the developed photoanode. Upon photon absorption at energies matching its bandgap, the CdSe compound generates charge carriers, which are subsequently transferred to the conduction band of LaNiO_3_. The excited electrons travel through the external circuit to the counter electrode, where H_2_ is generated as a result of the water splitting. Simultaneously, the holes generated in the LaNiO_3_ migrate to the valence band of CdSe, which in turn causes water separation, as described by Equations (1) and (2). The incorporation of CdSe not only improves visible light absorption but also reduces the recombination rate due to favorable band alignment, forcing the hole to move from the valence band of CdSe to that of LaNiO_3_, thereby improving overall charge separation and efficiency.

It should be mentioned that the mechanism discussed above is not new. In fact, it was well explained in an article published over a decade ago by Wang et al. [69], who stated that this is a semiconductor–semiconductor non-*p*-*n* heterojunction system. They highlighted the same conclusion, observing that when semiconductors A and B have similar band potentials, and the conduction band level of B is lower, photoexcited electrons from the conduction band of A can be transferred to B. A similar process occurs when the valence band of B is positioned lower, allowing holes to migrate from A to B. As a result, this charge transfer mechanism reduces the likelihood of electron–hole recombination, enhancing overall charge separation. Wang et al. [70] proposed an S-scheme heterojunction, designing a composite where CdSe was supported on carbon nitride nanorods. Photocatalytic experiments revealed that this composite achieved a hydrogen production rate of 20.1 mmol g^−1^ h^−1^, which was 4 times higher than only CdSe, and 19 times greater than pristine carbon nitride. An interesting piece of information that can be found in this research was the large R_ct_ reported for this composite, 9.73 × 10^5^ Ω. This could explain why the scientific community has reduced original research on CdSe-based materials. There has been a noticeable decline in publications after 2023, where such materials are used in water splitting. A recent study aimed at enhancing the activity of Cd by adding S to obtain CdS, which decorates hexagonal ZnO nanorods. However, a large R_ct_ was observed, close to 3000 Ω, confirming challenges in tuning the intrinsic properties of Cd [71]. Liu et al. [72] proposed a more complex composite incorporating SbS_3_, a narrow band gap composite, to decorate CdSe_x_S_1–x_, resulting in a core-shell quasi-one-dimensional nanomaterial. Although this photocatalyst exhibited an effective S-scheme heterojunction that enhanced electron–hole separation, resulting in a current density of 1.61 mA cm^−2^ at 1.23 V, its R_c_ remained high at 2178 Ω cm^−2^. Although CdSe-based materials possess a suitable band gap for water splitting (especially when compared to typical semiconductors with values above 3 eV), their high R_ct_ limits their potential for practical applications. The high charge transfer resistance is partly attributed to defects that trap charge carriers and reduce mobility. Therefore, doping remains one of the few viable strategies to enhance their efficiency.

### 3.3. NiWO_4_-Based Photoelectrocatalysts

**Figure 7:**
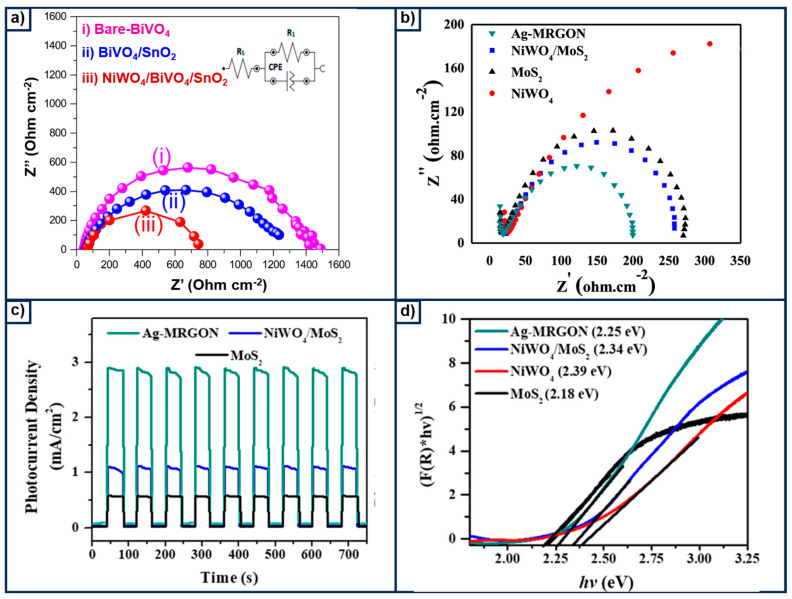
Characteristics for the composite, based on NiWO_4_. (**a**) EIS spectra for the photoanodes conducted at a bias of 1 V in 0.5 mol L^−1^ H_2_O_2_ (30%), reproduced under the terms and conditions of the Creative Commons Attribution (CC BY) license (http://creativecommons.org/licenses/by/4.0/, accessed on 16 January 2025) [76]. (**b**) EIS recorded at 0.2 bias voltage in 0.5 mol L^−1^ Na_2_SO_4_ supporting electrolyte. (**c**) The photoresponse of the NiWO_4_-based composites at a fixed potential of 1 V. (**d**) The Tauc’s plot for the composites, reproduced under the terms and conditions of the Creative Commons Attribution (CC BY NC-ND) [77].

Nickel tungstate (NiWO_4_) is another material highly recognized for its activity in achieving water splitting under visible light, owing to its practical band gap that provides sufficient energy to facilitate this reaction [19]. A research group, Zhu et al. [73], pioneered the development of a heterojunction based on a NiWO_4_ photoanode, dating back to the year 2013. The designed material shows a band gap ranging from 2.2 eV to 2.65 eV, capable of achieving an IPCE efficiency of 40.7%. Three years later, Do and coworkers [74] fabricated a self-assembled NiWO_4_–WO_3_ heteroepitaxy that showed an IPCE of 50%, and this improvement was ascribed to a better charge separation in the generated charge carriers at the interface of the material. The former was also confirmed by the small R_ct,_ which ranged from 105 to 86 Ω. Therefore, this material not only possesses an acceptable band gap but also achieves R_ct_ values that are optimal for water splitting, critical factors that often limit the performance of traditional semiconductors and CdSe-based materials. From that point on, a large amount of research has been published in this direction. Hosseini et al. [75] reported the fabrication of NiWO_4_ nanoparticles supported on graphene, which displayed a band gap from 2.7 eV to 3.2 eV, and this increment was attributed to the effect of grain reduction. This finding is crucial, as grain size can be easily tuned by adjusting synthesis parameters. This opens up new avenues for material engineering, enabling the optimization of photocatalytic and electronic properties through controlled nanostructuring and process tailoring. The FESEM images reveal changes in both morphology and particle size. For instance, bare BiVO_4_ particles exhibit an average size of approximately 500 nm, which decreases to below 300 nm upon the addition of NiWO_4_ and SnO_2_. This reduction in particle size leads to smaller grain sizes, which can ultimately enhance electron transfer during the photoelectrocatalytic process. To further understand the photoelectrochemical properties of NiWO_4,_ Shaddad et al. [76] conducted comprehensive research to elucidate the role of this compound when mixed with BiVO_4_ and SnO_2_ and then tested as a photoanode. Surprisingly, the materials exhibited an enhanced OER, which demonstrated a charge separation efficiency of 30%. To gain a deeper understanding of this effect, an electrochemical impedance spectroscopy (EIS) test was conducted, and this is illustrated in Figure 7a.

As is well recognized, the arc diameter of the depressed semicircle is related to interfacial charge transfer resistance and also the surface recombination process. To this end, the authors proposed an equivalent electronic circuit consisting of a series of resistance elements connected to a parallel arrangement of resistance and a constant phase element. In the case of the samples containing NiWO_4_, the diameter was smaller, below 800 Ω cm^−2^, which corroborates the better performance of these materials in conducting the OER. It should be noted that these R_ct_ values are significantly lower when compared to those reported for CdSe-based photoelectrocatalysts, where values exceeding 2000 Ω cm^−2^ have been observed. In the same direction, Hendi et al. [77] designed a Z-scheme MoS_2_/RGO/NiWO_4_ (Ag-MRGON) heterostructure modified with Ag nanoparticles. This composite not only exhibits exceptional performance in PEC water splitting but also achieves a remarkably low onset potential of 0.61 V, significantly lower than the 0.83 V observed for NiWO_4_. The EIS results revealed that Ag-MRGON (see Figure 7b) has the smallest arc radius, which indicates facile charge transfer due to a synergistic effect between the Z-scheme heterojunction and the surface plasmon resonance of the added Ag nanoparticles. The former is further corroborated by the improved photocurrent response and small band gap, as shown in Figure 7c,d. Apart from the well-studied NiWO_4_, Li et al. [78] proposed a WO_3_/W nanotube array fabricated via a simple electrochemical anodization process and tested it as a photoanode in water splitting. The band gap of the modified electrode was quantized by a Tauc’s plot, revealing a band gap of 2.35 eV, which is smaller than the conventional WO_3_/W electrode, at 2.45 eV. Although this material was initially proposed for use in water splitting, the authors found good performance for methanol oxidation when this alcohol was added as a hole scavenger. The reported efficiency of this material during the PEC activity was 5.23% under an irradiation source, with a wavelength of 420 nm and 15 mW cm^−2^. Compressive electrochemical research conducted on NiWO_4_ electrodes reveals an anodic peak appearing close to 1.23 V, which is due to the oxidation of Ni^+2^ and W^+6^ states, improving the charge transfer mechanism at the electrode–electrolyte interface [79]. This phenomenon was also detected during the CA test, where the voltage–time curve had an increasing tendency profile even when exposed to a large time, 20,000 s, which is typical behavior for electrode reconstruction. The stability of NiWO_4_ anodes is one of the big barriers, which is why Li and coworkers [80] combined this with NiSe_2_, employing a two-step hydrothermal method, supported on nickel foam. Transmission Electron Microscopy (TEM) and High-Resolution Transmission Electron Microscopy (HRTEM) show the successful selenization of NiWO_4_/NF, forming a core-shell structure with a lattice parameter of 0.379 nm (110) NiSe_2_. A chronoamperometry test was conducted at a cell voltage of 1.6 V for 15 h, observing the typical behavior of a stable heterostructure. Similar works have also been commenting on this good stability [81], making this material promising, like electrocatalysts and photoelectrocatalysts. From these observations, it can be deduced that the band gap of NiWO_4_-based photocatalysts can be tuned within a range of approximately 2.25 eV to 3.2 eV, depending on the synthesis method and the elements incorporated into its matrix. It is important to note that an ideal band gap for practical applications lies above 1.4 eV but below 3 eV, to ensure efficient absorption of visible light while minimizing UV light. Therefore, NiWO_4_ photocatalysts may offer greater promise than CdSe-based materials in terms of both tunability and visible-light-driven performance.

### 3.4. Graphitic Carbon Nitride-Based Photoelectrocatalysts

**Figure 8:**
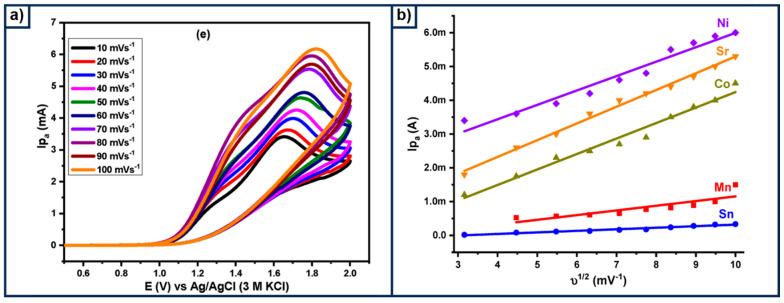
Electrochemical characterization in 1 mol L^−1^ KOH and at different scan rates. (**a**) The CV curve for g-C_3_N_4_-NiWO_4_. (**b**) The relationship between the anodic peak (I_pa_) and the scan rate square root v^1/2^ for all the modified g-C_3_N_4_/Metal WO_4_. Reproduced under the terms and conditions of the Creative Commons Attribution (CC BY) license (https://creativecommons.org/licenses/by/4.0/) [84].

Graphitic carbon nitride (g-C_3_N_4_) is a carbonaceous material that has been proven to enhance the charge transfer in modified composites. Its unique physicochemical properties make it a promising candidate for photoelectrocatalytic applications [82]. For instance, Rajaitha et al. [83] conducted a detailed analysis of the effect of g-C_3_N_4_ composition on TiO_2_ nanomaterials. The graphitic material was obtained by mixing melamine and ammonium sulphate and then thermally treated at 550 °C. Afterwards, this composite was dispersed in an H_2_SO_4_ electrolyte solution, and TiO_2_ powder was added under sonication. The slurry was maintained under agitation at a temperature of 80 C for 1 h. The pH of the solution was adjusted with ammonia until it reached a pH of 7, and finally, the sample was recovered by centrifugation, washed, and dried. The absorption studies revealed that an increase in g-C_3_N_4_ provokes a displacement at a longer wavelength, while the determined bandgap value decreases from 2.82 eV to 2.73 eV as the g-C_3_N_4_ increases. Moreover, the water splitting tests confirmed the versatility of the g-C_3_N_4_/TiO_2_ composite, which delivers a photocurrent of 142.7 μA cm^−2^ at 1.23 V as a result of the recombination rate reduction. It should be noted that this photocurrent density is very close to that reported for pristine TiO_2_, 0.17 mA cm^−2^, making the material a promising candidate for further photoelectrocatalytic applications [62]. With the idea of reducing energy consumption during electrochemical water splitting, Ashfaq explored [84] the modification of g-C_3_N_4_/TiO_2_ with different metal tungstates. In this case, the g-C_3_N_4_ was obtained by mixing melamine and cyanuric acid sources, while the modified one with metal tungstates was obtained by adding Na_2_WO_4_·2H_2_O to the obtained g-C_3_N_4_ and submitted to a hydrothermal process, using only a NaOH solution. The g-C_3_N_4_/WO_4_ was modified by adding Ni, Co, Mg, Sn, and Sr. Figure 8a presents the CV at different scan rates for g-C_3_N_4_/NiWO_4_ and other materials, observing an increase in the anodic current density as the scan rate is increased. Although the onset potential for all the materials was above 1 V vs. NHE for all composites, the authors stated that the addition of the carbonaceous composite decreased this. The effect of the intermetallic composite can be observed in Figure 8b, where the g-C_3_N_4_/NiWO_4_ composite delivers the highest current densities at any value of v^1/2^. It should be mentioned that the authors did not include the anodic current for the composite g-C_3_N_4_/MnWO_4_ at a very low scant rate (Figure 8b), which might be due to the low electroactive presented by this material.

Murugan et al. [85] conducted detailed research trying to design a heterojunction material aimed at enhancing hole mobility through a two-step synthesis process. First, g-C_3_N_4_ nanostructures were developed by calcinating a mixture of melamine and ammonium sulphate at 530 °C. Then, the resulting composite was combined with polyvinylpyrrolidone sodium dodecyl sulphate and titanium (IV) isopropoxide, with the pH adjusted using an ammonia solution. Finally, the collected sample was calcinated at 450 °C for 30 min. FESEM and TEM demonstrated the formation of g-C_3_N_4_ with a nanosheet structure, while TiO_2_ appeared as nanospheres. Surprisingly, the photocatalyst exhibited a band gap of 2.74 eV, which is significantly smaller than that of TiO_2_, typically measured at 3.2 eV. Furthermore, this band gap is similar to that reported for NiWO_4_ photocatalysts, making these materials promising candidates for PEC cells. Nonetheless, after photoelectrochemical characterization, it was observed that the sample containing 20 wt.% g-C_3_N_4_ delivered only a current density of 72.3 μA cm^−2^ at 1.23 V, which is significantly lower than the values reported for TiO_2_. Due to the low efficiencies of nitride (g-C_3_N_4_), several strategies have been adopted to enhance current density and kinetics in water oxidation. In this sense, Yang et al. [86] reported the TiO_2_ modification with cobalt-substituted polyoxometallate (Co-POM) by a two-step process. Although the study described a successful photoanode modification, where TiO_2_ was covered by a well-defined layer of Co-POM, the PEC tests revealed low efficiency, achieving only 0.42 mA cm^−2^ at 1.23 V. The low performance might be partly attributed to the large R_ct_ value, 953.9 Ω, calculated from the EIS data and fitted using a simplified Randles circuit. The latter considerably reduces the kinetics of water oxidation, driving the scientific community to continuously seek better prospects. Yang et al. [87] studied the modification of graphitic carbon nitride nanosheets with Ti-doped hematite and Ni-doped CoPcocatalysts (Ti-Fe_2_O_3_/g-C_3_N_4_/Ni-CoP) to form a type-II heterojunction. The resulting material exhibited a photocurrent density of 2.01 mA cm^−2^ at 1.23 V. The main disadvantage of this approach is the complex and time-consuming synthesis process, which involves the formation of g-C_3_N_4_ through an urea annealing process, followed by modification with Ni-doped Co_3_O_4_ (NCO). This is then followed by a phosphidation step, where the CN-supported NCO was covered with Ni-doped CoP (CN/NCP). Afterwards, Ti-Fe_2_O_3_ was grown on a fluorine-doped tin oxide (FTO) substrate, and the CN/NCP was deposited by spin coating. A more formidable strategy was reported by Sundararaj [88], in which NiWO_4_ and g-C_3_N_4_ were combined to form a heterojunction. First, g-C_3_N_4_ was fabricated by the thermal polycondensation of melamine and ammonium sulphate at 550 °C, followed by washing with ethanol. The NiWO_4_/g-C_3_N_4_ was then prepared by exfoliating g-C_3_N_4_ and incorporating NiWO_4_ through a solvothermal process, using hexamethylenetetramine, NiCl_2_ 6H_2_O, ammonium fluoride, Na_2_WO_4_ 2H_2_O, and NaOH. The data obtained from UV–visible experiments reveal that the band gap of the composite was easily tuned by adjusting g-C_3_N_4_ content, ranging from 2.33 eV to 2.79 eV. Similarly, the PEC measurements showed that the R_c_ of the composite varied according to the g-C_3_N_4_ weight fraction, obtaining a very small value of 8.33 Ω. at 15 wt.% of g-C_3_N_4_. Also, this former composite delivers the highest photocurrent density, with a value of 167.81 mA cm^−2^, which was mainly attributed to its effective charge separation, along with its low charge carrier recombination. Moreover, Mott–Schottky analysis confirmed that the final composite behaved as a *p*-type semiconductor, indicated by its negative slope, demonstrating that NiWO_4_ governed the material’s intrinsic properties. Notably, the composite exhibited a remarkably low overpotential of 0.048 V to achieve the standard 10 mA cm^−2^ for the HER, and a small Tafel slope of 43 mV dec^−1^, highlighting its excellent electrocatalytic performance. Based on the above observations, it can be concluded that g-C_3_N_4_ alone does not possess the required properties for efficient water splitting. Modifications to its structure, such as incorporating other semiconductors or metal elements like Ni, Co, or Mg, are necessary. Although some materials exhibit high current densities at 1.23 V, their fabrication processes are too complex, which limits their widespread application in practical scenarios.

### 3.5. Fe_2_O_3_-Based Photoelectrocatalysts

Hematite (α-Fe_2_O_3_) is a metal oxide that has been considered a photoanode due to its good chemical stability and low cost of production, along with an adequate band gap of 2.2 eV [89,90]. As mentioned above, the value of the band gap plays a critical role in determining the ability of the material to absorb light within the visible spectrum. Nonetheless, it is well-recognized that pristine hematite has poor performance in charge transfer separation and low conductivity [91,92]. In this regard, a lot of proposals have been implemented to try to face this limitation, including doping, introducing advanced structures, stimulating oxygen vacancies, surface modification, and others [93,94,95]. Earlier strategies considered the hematite modification with some metals or semiconductive materials like Ti, obtaining current densities of 0.5 mA cm^−2^ at 1.23 V [96]. The main drawback of these photoanodes was their large charge transfer resistance, obtaining values even higher than 1600 Ω, as was observed from the EIS plot, which decreased efficiency. This value is quite similar to those observed in BiVO_4_ and CdSe-based photocatalysts, which exhibit low performance in water splitting when used without modification. In this context, Yanming et al. [97] developed α-Fe_2_O_3_/Au/TiO_2_ to investigate water oxidation under light exposure. The test demonstrated a better performance of this electrode, reaching a current density of 1.05 mA cm^−2^ at 1.23 V, and this was ascribed to reconstructed interfaces that help in reducing bulk and surface recombination, which was corroborated by the small R_ct_ trap, with a value of 447.8 Ω cm^−1^. Li et al. [98] followed a similar pathway, where Fe_2_O_3_ was doped with ZnO and deposited on the surface of modified hematite. From the LSV, it can be observed that Fe_2_O_3_/Zn-Fe_2_O_3_-120 μL has better performance in water splitting, 1.34 mA cm^−2^ at 1.23 V, which can be directly related to better light absorption and a low band gap. This was confirmed by the UV–Vis measurements, which showed a band gap ranging from 1.9 to 1.93 eV, depending on the composition of the material. Moreover, the MS analysis measured in 1 mol L^−1^ NaOH solution in the dark at a fixed frequency of 1000 Hz showed a positive slope, which is the typical behavior of *n*-type semiconductors with a donor density of 7.94 × 10^19^ cm^−3^. An important finding highlighted in the manuscript addresses the correlation between R_ct_ and capacitance attributed to surface states (C_ss_). It states that a reduction in R_ct_ and an increase in C_ss_ indicate that hole transfer during water oxidation occurs through surface states. Therefore, α-Fe_2_O_3_-based materials are positioned as promising semiconductors for the development of PEC cells, due to their high current density at 1.23 V and tunable band gap ranging from. Li and coworkers [99] developed an interesting process where hematite was modified by adding lactic acid during the in situ hydrothermal growth on the FTO substrate. The improvement in photocurrent density (1.5 mA cm^−2^ at 1.5 V vs. 0.5 mA cm^−2^ for the untreated hematite) was mainly attributed to the favored oxygen vacancies and the morphology-like island pattern created when this acid was added. The latter was corroborated by the research group using an EIS-fitted electronic circuit, where the charge transfer resistance shows a decrease in all the samples treated with lactic acid, from 607 Ω to 137 Ω for the optimal composition. The cocatalysts approach was adopted by Chong et al. [100], who synthesized CoOOH/Fe_2_O_3_ photoelectrocatalysts with promising efficiency. One particular aspect was the development of CoOOH using a two-step synthesis process. In the first step, the authors grew Co(OH)_2_ on a Fe_2_O_3_ photoanode by mixing CoCl_2_ in an ethanol solution, followed by autoclave treatment. The second step introduced the following novel approach: the as-prepared sample was further treated in a 0.5 mol L^−1^ H_2_O_2_ solution and subsequently thermalized at 150 °C. This unconventional method is rarely seen in typical synthesis processes, adding a unique dimension to their work. Even more surprising was the current density achieved by this treated sample, which reached an impressive 1.92 mA cm^−2^ at 1.23 V, approximately 2.6 times higher than that of Fe_2_O_3_. The improvement was ascribed to the capacity of CoOOH species to passivate the Fe_2_O_3_ surface while simultaneously acting as active sites for water splitting. In terms of the band gap, the sample reached approximately 2.1 eV, which is nearly identical to the value measured for Fe_2_O_3_. Moreover, a significant difference was observed in the R_ct, trap,_ which decreased by a factor of 2.7 following the incorporation of the CoOOH layer.

**Figure 9:**
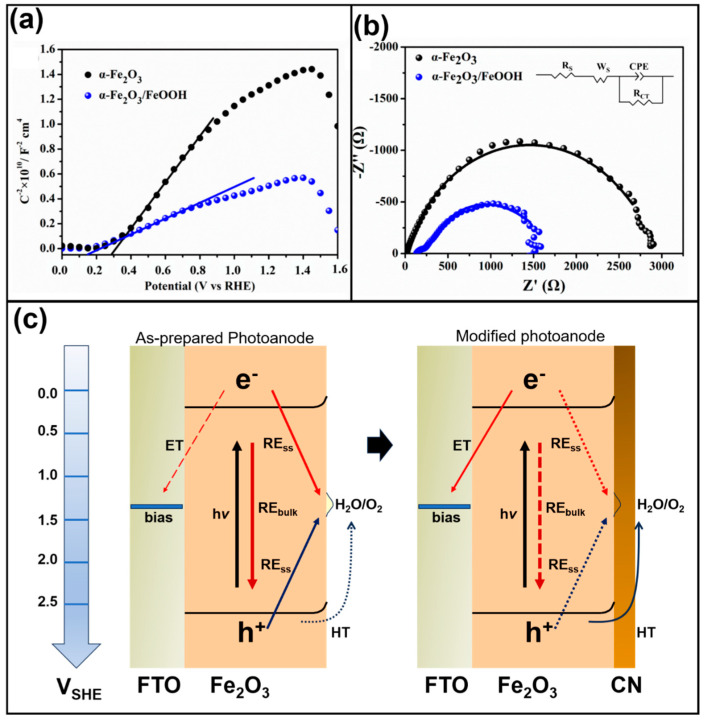
(**a**) The Mott–Schottky diagram of Fe_2_O_3_ and α-Fe_2_O_3_/FeOOH measured at 1 kHz under dark conditions. (**b**) The EIS spectra for Fe_2_O_3_ and α-Fe_2_O_3_/FeOOH 1 mol L^−1^ NaOH at 0.5 V. Reproduced under the terms and conditions of the Creative Commons Attribution (CC BY) license (https://creativecommons.org/licenses/by/4.0/) [89]. (**c**) The schematic diagram for the charge transfer mechanism in the hematite photoanode and the hematite modified electrode with graphitic carbon nitride. ET, HT, RE, and SS are electron and hole transfer, recombination, and surface trap sites, respectively. Copyright 2024 Elsevier, reproduced with permission from reference [103].

The surface passivation effect has been a topic of discussion, and several scientists have been trying to explain this. Xie et al. [101] developed an α-Fe_2_O_3_ photoanode modified with a Co-based hole transport layer to study these phenomena. For instance, Figure 9a,b show the photoelectrochemical response of Fe_2_O_3_ and Fe_2_O_3_/FeOOH in 1 mol L^−1^ NaOH. Herein, it can be observed that both materials exhibit a positive slope, indicative of an *n*-type semiconductor. Notably, Fe_2_O_3_/FeOOH has a smaller flat band potential of 0.14 V, which is mainly ascribed to the presence of FeOOH. This advantage was confirmed by the EIS diagram, revealing nearly half the resistivity compared with the Fe_2_O_3_ electrode. The Fe_2_O_3_/ZnO/CoTCPP exhibited higher photocurrent density with a value of 2.16 mA cm^−2^ at 1.23 V, making it one of the most effective Fe_2_O_3_-based photoelectrodes reported. However, in this material, the reaction can be hindered by phenomena such as charge recombination (this is represented in the diagram, see Figure 9c, by solid lines that originate from the electron and the hole), short carrier lifetimes, short lives, and the need for high overpotentials. To face this issue, graphitic carbon nitride or another cocatalyst can be incorporated, helping in hole transport. This additional layer can act as a passivating surface, mitigating the charge recombination in the semiconductor (represented by a dashed line that originates from the electron and hole). Yin et al. [102] discussed this from a mechanistic point of view in the BiVO_4_ photoanode modified with a ferrihydride layer. They stated that photoinduced holes reach the BiVO_4_ surface, where they can either react with water or recombine with back electrons. The ferrihydride layer plays a vital role, as it reduces charge recombination by inducing significant band bending through surface passivation.

A more refined approach was implemented by Jeon et al. [103], who modified α-Fe_2_O_3_ by depositing a 5 nm thickness of g-C_3_N_4_ and metals, such as Ag, Fe, and Co, via the evaporation method. Although this alternative is more time-consuming and costly compared with the work proposed by Li et al. [99], it helps us to understand how the hematite structure can be tuned to enhance its photoconversion efficiency and, in this way, reach efficiencies above the actual 7% observed. The main idea of the mentioned work was creating a passivation layer that, in turn, results in better charge transfer and low recombination. As expected, the bare hematite electrode delivers low photocurrent density, 0.5 mA cm^−2^ at 1.23 V. However, modifying the sample with a 5 nm g-C_3_N_4_ layer doubles the current density at the same potential. Surprisingly, the incorporation of Co into the α-Fe_2_O_3_/g-C_3_N_4_ composite increases the current density five-fold, demonstrating a significant enhancement in performance, mainly attributed to the capacity of more light absorption.

**Figure 10:**
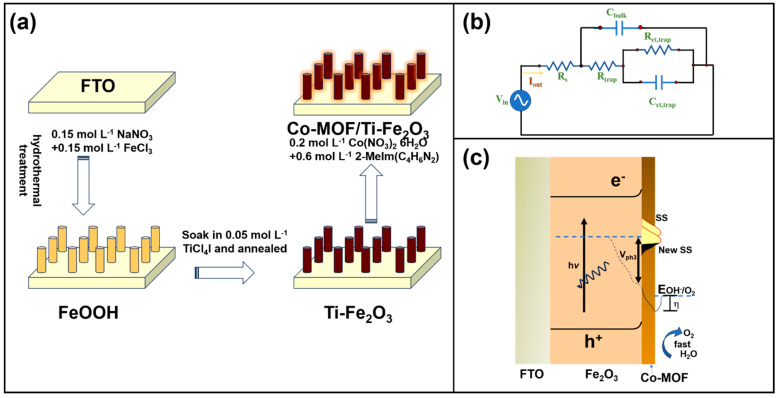
(**a**) Steps of the procedure for the Co-MOF/Ti-Fe_2_O_3_ photoanode fabrication. (**b**) The EIS electronic circuit to fit the experimental EIS curve. (**c**) The proposed mechanism for water oxidation on the developed anode. Copyright 2023 Elsevier, reproduced with permission from reference [104].

An outstanding proposal was to fabricate a photoanode coated with a Co-based metal-organic framework (Co-MOF) and Ti-doped Fe_2_O_3_ [104]. Figure 10a illustrates the steps for the anode fabrication, involving hydrothermal and thermal treatment, for the successful obtention of the Co-MOF/Ti-Fe_2_O_3_ electrode. In this study, researchers conducted a detailed analysis to elucidate the main mechanism of water oxidation. The adopted electronic circuit, see Figure 10b, accounts not only for the capacitive behavior of the photoelectrochemical process but also for the intrinsic capacitance of the bulk hematite on the surface electrode. The EIS plot suggested low R_trap_ and R_ct,trap_ values for the Co-MOF/Ti-Fe_2_O_3_ electrode in comparison with the Fe_2_O_3_ sample. For example, at 1.2 V, the Co-MOF/Ti-Fe_2_O_3_ electrode delivers a R_ct,trap_ of approximately 220 Ω cm^−2^, whereas Fe_2_O_3_ exhibited 1350 Ω cm^−2^. Additionally, Figure 10c depicts the proposed water oxidation mechanism for this material (Fe_2_O_3_ covered with a Co-MOF layer) which is similar to that in [103], but they also highlighted the crucial role of the Co-MOF in reducing the overpotential and acceleration of water splitting, as well as the influence of surface states (SSs) on the reaction’s initial potential. It is observed that the creation of a new surface state shifts the V_on_ at moderate values of 0.80 V_RHE_ after the addition of Co, which is responsible for the faster kinetic O_2_ formation at low overpotential.

**Figure 11:**
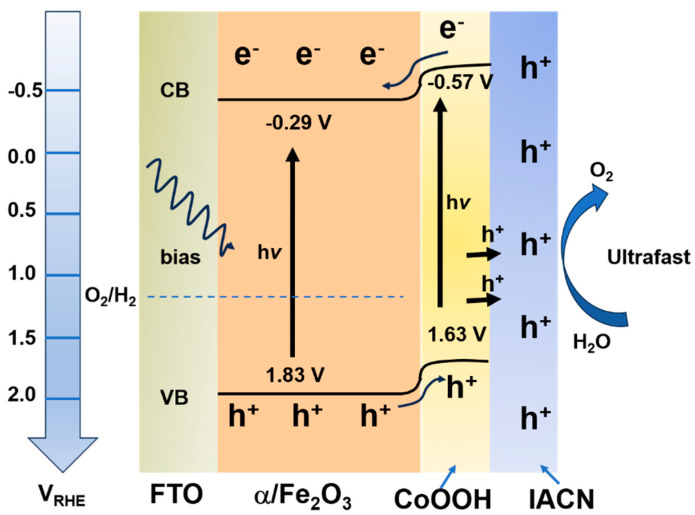
Reported diagram of charge carriers and water oxidation on IACN/CoOOH/Fe_2_O_3_. Copyright 2024 Elsevier, reproduced with permission from reference [107].

A similar approach was explored, but in this case, Fe_2_O_3_ was doped with Sn and further modified with amorphous cobalt oxide [105]. This modification introduced a novel aspect, enabling the photoanode to reach a current density of 1.4 mA cm^−2^ at 1.23 V, surpassing the 1.01 mA cm^−2^ reported in [104]. This advantage might be due to the ability of cobalt oxides (Co^2+^, Co^3+^) to modify the oxygen vacancies, which in turn enhances charge carriers. This was further confirmed by the low R_ct,trap_ value at 190 Ω cm^−2^, which was estimated by fitting the EIS response with the electronic model proposed in [104]. Although the aforementioned photocatalysts demonstrate acceptable performance, considering the low cost of the chemical elements used in the synthesis process, their efficiency still needs improvement for the widespread application of photoelectrocatalysts for H_2_ and O_2_ generation. In this regard, Bai et al. [106] implemented an innovative strategy to address the challenges of Fe_2_O_3_ electrodes by doping them with Ti and subsequently adding an amorphous PdCoP layer. The photoanode fabrication process was quite similar to those reported above, where the FTO was placed in an autoclave with the Fe and Ti precursors and then thermally treated at temperatures above 550 °C. Afterward, an aqueous solution containing PdCl_2_ and Co(NO_3_)_2_ 6H_2_O was added to the resulting composite, while the NaH_2_PO_4_ was injected into the tube furnace where the electrode was placed. The photoelectrochemical test reveals that this novel photoanode is capable of delivering a current density of 2.82 mA cm^−1^ at 1.23 V, which is superior to those previously mentioned. One notable aspect is that these authors used a simple electronic circuit consisting of two loops, each containing a parallel resistor–capacitor network, interconnected with a resistor that simulates the electrolyte resistance. Although they estimated parameters using different models from the EIS, it was clearly observed that the suppressed semicircle had a small diameter, indicating a low R_ct_, 123.4 Ω. Recent work has revived the concept of using CoOOH to treat the hematite compound; however, this time, a novel addition was introduced, a hole transfer layer (HTL) that seeks to solve the charge transfer between this and the commonly employed catalysts [107]. To this end, metal–organic coordination was utilized in conjunction with Co and Ni species to obtain the catalysts, while CoOOH served as the HTL. X-ray photoelectron spectroscopy (XPS), along with Fourier Transform Infrared Spectroscopy (FTIR) results, confirmed the successful formation of each layer, as evidenced by the presence of C-C, O=C-O, C=N, and M-N bonds. These unexpected photoelectrochemical measurements show the high efficiency of this material, attaining a current density of 2.02 mA cm^−2^ at 1.23 V, which is superior to most reported photoanodes. The author stated that CoOOH has better performance as a hole extraction layer, improving water oxidation kinetics. Furthermore, EIS confirms the advancement of this material (simulated using the same model presented in Figure 10b), as demonstrated by the R_ct_ value of 101.8 Ω cm^2^. Figure 11 summarizes the parameters determined from the experimental measurements and the expected mechanisms in water oxidation. The conduction band for Fe_2_O_3_ was −0.29 V while the CoOOH has −0.57 V. Their respective valence bands are 1.83 V and 1.63 V, indicating a type II heterojunction band structure. Additionally, the electron transfer from the IACN layer occurs through bridges formed by a coordination bond.

Therefore, it can be noted that Fe_2_O_3_-based photoelectrocatalysts are promising candidates for constructing PEC cells. However, they require modification with additional layers, such as CoOOH, FeOOH, or metal–organic frameworks, to enhance their performance. The main drawback lies in the complex synthesis process, which typically involves multiple steps, posing challenges for large-scale production.

### 3.6. Other Promising Photoanode Candidates

**Figure 12:**
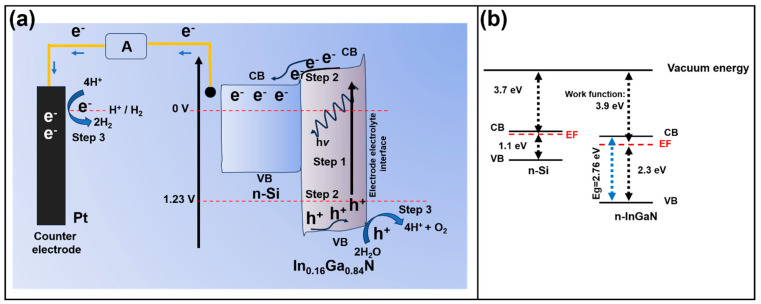
(**a**) Scheme of the PEC, and (**b**) band alignment of the n-InGaN/n-Psi, where VB, CB, and EF are the valence band, conduction band, and the Fermi level, respectively. Copyright 2021 Elsevier, reproduced with permission from reference [108].

Further studies have attempted to modify the design of structural nanomaterials to enhance water-splitting efficiency. A research group led by Chen et al. [108] explored the effect of InGaN structures deposited on the transition from a planar to a pyramidal texture on Si(100) surfaces as anti-reflection and light trapping. The preferential texture was developed through wet chemical etching, with consecutive treatment of 20 wt.% NaOH, 5 wt.% NaOH + 5 vol.% 2-propanol, 20 wt.% HCl, and finally 10 wt.% HF, whereas the InGaN nanowires were grown via plasma-assisted molecular beam epitaxy. Figure 12a presents the PEC cell process proposed by the authors. Step 1 involves light absorption corresponding to the material’s band gap. Step 2 represents the movement of photo-generated electrons and holes. Step 3 is associated with surface reactions, specifically H_2_ and O_2_ production. InGaN is responsible for the generated charge carriers and directs holes to the surface to achieve water oxidation, and due to the slight difference in energy level, a charge transfer between this compound and the tuned Si is expected. Meanwhile, Figure 12b depicts the changes occurring in n-Si and n-InGaN layers regarding band alignment, indicating that the difference between the EF and the VB of InGaN is close to 2.3 eV. Meanwhile, the work function was calculated by subtracting the cut-off energy (17.32 eV) from the used photon energy (21.22 eV), resulting in a value of 3.9 eV. Although the authors conducted electrochemical measurements, such as EIS and CA, they did not report the photocurrent density for these materials. However, the EIS results revealed a high charge transfer resistance of 800 kΩ, which is significantly greater than that observed in traditional semiconductors and even CdSe-based materials. This presents a major challenge for the application of these materials.

**Figure 13:**
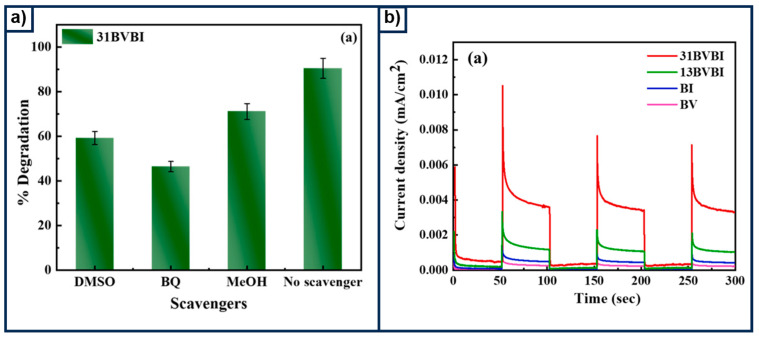
(**a**) The effect of dimethyl sulfoxide (DMSO), benzoquinone (BQ), and methanol (MeOH) as scavengers for the degradation of tetracycline. (**b**) Transient photocurrent at 1.23 V vs. RHE. Reproduced under the terms and conditions of the Creative Commons Attribution (CC BY) license (https://creativecommons.org/licenses/by/4.0/) [109].

An outstanding strategy has been explored by scientists who have studied materials with multipurpose applications. A BiVO_4_/BiOBr composite, synthesized by the hydrothermal method, was tested as a photocatalyst for tetracycline degradation and water oxidation [109]. The researchers stated that the growth of BiOBr on the surface of BiVO_4_ enhanced interfacial contact, which reduces charge recombination and improves solar light absorption. The latter was corroborated by a photoluminescence curve, where the proposed composite showed low signal intensity in comparison with BiVO_4_, indicative of less carrier recombination. Surprisingly, the material presented a higher degradation rate when no scavengers were used, see Figure 13a, indicating that the mechanism for degradation is mainly via reactive oxygen species. However, chopped LSV conducted at 1.23 V showed that BiVO_4_/BiOBr had appreciable current densities when it was excited to simulated light, with the latter explaining its versatility in achieving water oxidation (Figure 13b). However, the test revealed a low photocurrent density of 0.2198 mA cm^−2^, which is nearly the same value obtained for TiO_2_ modified with a CoO-Co_3_O_4_ layer, at 0.23 mA cm^−2^. This issue was related to the high R_ct_, with observed values above 83 kΩ, depending on the molar ratio.

Bismuth oxycarbonate (Bi_2_O_2_CO_3_) nanosheets were also utilized to construct a heterojunction with Ni(OH)_2_ structures and used as a photoanode in water splitting [110]. The composite was synthesized following the facile hydrothermal steps, where bismuth nitrate and nickel nitrate were mixed in an ethylene glycol–deionized water solution. The mixture was modified by adding urea and polyvinylpyrrolidone, followed by thermal treatment in a Teflon line at 180 °C. The band gap of the sample was determined by solving the Kubelka–Munk function for the reflectance spectra, observing a direct band gap of 3.76 eV, which is slightly higher compared to the unmodified Bi_2_O_2_CO_3_ (3.43 eV). Nonetheless, Bi_2_O_2_CO_3_@Ni(OH)_2_ delivers a photocurrent density of 5.87 mA cm^2^ at 1.2 V vs. Ag/AgCl, nearly five times higher than Bi_2_O_2_CO_3_. This enhancement is attributed to the faster charge transfer (R_ct_ 5.27 Ω) enabled by the incorporation of Ni(OH)_2_, resulting from adequate band gap alignment. Additionally, the authors emphasize the significance of the flower-like structure, which enhances surface–electrolyte interactions.

Another proposal explored the effect of extremely low input light energy in a Molybdenum plate-like structure doped with tungsten(VI) oxide and deposited on Sb_2_S_3_ [111]. The PEC test demonstrated the ability of this composite to sustain a photocurrent of 0.42 mA cm^−2^, almost 20 times higher in comparison with the unsupported one. This enhancement was attributed to a broader band gap, allowing for greater photon absorption, as well as improved electron–hole separation efficiency. Moreover, long-term stability experiments have revealed a steady current, which is the result of low corrosion, making this material promising for applications where low light is available. CsPbBr_3_ perovskite material has also been considered as a candidate for producing water oxidation due to its optoelectronic characteristics, making it promising not only for photovoltaic applications but also for light-emitting applications [112,113]. Gong and coworkers [114] used this halide perovskite material doped with Pd, obtaining CsPbBr_3_, which was the most active in the water oxidation. The sample achieved a current density of 2.07 mA cm^−2^ at 1.23 V, demonstrating remarkable stability even after 1200 s. Notably, when noble metals are utilized, their role as a doping agent makes this a viable strategy, as only a minimal amount of the precious element is required.

Table 1 summarizes key reported materials exhibiting activity in water oxidation. From this, it can be inferred that the hydrothermal method is the most commonly employed synthesis process for producing photoelectrocatalysts. In some cases, multiple techniques are required to obtain the desired material, which introduces technical challenges and increased costs that must be carefully considered when scaling up production. The most active material for the OER was WO_3_/BiVO_4_/Cu_2_O, followed by CN-FeNiOOH-CoOOH, which delivered 5 mA cm^−2^ and 3.5 mA cm^−2^, respectively. To gain insight into the origin of the acceptable photocurrent densities, a review of parameters, such as the band gap and charge transfer resistance (Rct), was carried out. In the case of the CN-FeNiOOH-CoOOH, a band gap of 2.1 eV and RCT below 1 kΩ were observed, which is notably low considering that this material incorporates g-C_3_N_4_. For the WO_3_/BiVO_4_/Cu_2_O composite, the band gap of each layer was determined to be 2.71 eV for WO_3_, 2.52 eV for BiVO_4_, and 1.99 eV for Cu_2_O. It should be mentioned that the WO_3_/BiVO_4_ heterojunction only delivered a photocurrent density of 2 mAcm^−2^ at 1.23 V. However, after the addition of Cu_2_O, this photocurrent increased significantly to 5 mAcm^−2^. The effectiveness of this improvement was evident from using a Nyquist plot, where the three-layer composite exhibited the smallest arc diameter, indicating reduced charge transfer resistance. The calculated values for the Rct were 111.2 Ω and 61.81 Ω for WO_3_/BiVO_4_ and WO_3_/BiVO_4_/Cu_2_O, respectively.

An intriguing approach that merits further investigation is theoretical simulation, given the valuable insights that it can provide. For instance, Liu et al. proposed a shear-strained Pd structure suspended in CuO and employed density functional theory to model the system. Their goal was to elucidate the reaction mechanisms involved in nitrate reduction to NH_3_ by calculating the free energy of the reaction steps. This pathway is particularly important, as it offers a promising route for large-scale hydrogen production. The results indicate that the *NO_3_ adsorption step is spontaneous in the defective material, highlighting the potential of engineered defects in enhancing catalytic activity [124].

## 4. Outlooks, Perspectives, and Strategies

Although some research groups have achieved acceptable efficiencies with the developed anodes, further research is needed to optimize their performance. A deeper analysis reveals that, in some cases, the synthesis methods for photoactive materials and the fabrication processes for photoanodes are not truly scalable. Additionally, most tests are conducted under controlled conditions, using high-purity water and a continuous light source, which raises concerns about real-world applicability. The presence of chloride ions or dissolved solids, such as calcium, in natural water sources could significantly impact the efficiency of photoelectrocatalysts. Moreover, temperature remains an underexplored factor. In some regions, temperatures exceed 40 °C, which can accelerate material degradation and must be carefully considered. It is expected that some materials could serve a dual purpose, simultaneously producing H_2_ and O_2_ while facilitating the oxidation of harmful molecules, such as dyes. However, significant research is still needed to identify the optimal combinations of active elements that work synergistically to enhance the intrinsic properties of photoelectrocatalysts. Despite considerable efforts having been made in the area of photocatalyst design, more theoretical calculations are required to fully understand the mechanism involved in photo-induced water splitting. The latter not only reduces the time required for research but also lowers laboratory costs by minimizing the use of reagents, thereby reducing environmental pollution associated with synthesis. Machine learning and genetic algorithms can play a crucial role in uncovering these mechanisms, but only if they are systematically applied. This approach has been extensively adopted in more mature technologies, such as batteries, to assess the effects of various parameters on the state of health of the battery. However, it can also be extended to reveal the intrinsic properties of photoelectrocatalysts. Before this, a multidisciplinary research group is needed to generate raw data from various proposed materials, which can then be used to train models or optimize equations for better predictive accuracy.

## 5. Conclusions

Society is facing energy challenges due to increasing power demands, making the exploration of alternative fuels like hydrogen a sustainable option. Photoelectrocatalysts can facilitate the transition from petroleum-based energy technologies by harnessing sunlight to convert the chemical energy of water into a more reliable fuel source. However, several barriers must be overcome first, including the development of materials with an appropriate bandgap for visible light absorption, reducing charge carrier recombination, improving stability, and designing low-cost photoanodes. After an analysis of the literature, it appears that the sole use of common semiconductors, such as TiO_2_, ZnO, and SnO_2_, is not sufficient to achieve practical volumes of H_2_ and O_2_. Therefore, tuning the intrinsic properties of these base materials by incorporating other elements can help modify the band structure and enhance light absorption. To this end, Fe_2_O_3_, g-C_3_N_4_, BiVO_4,_ and FeNiOOH photoanodes have emerged as potential candidates to boost water oxidation, demonstrating more favorable kinetics and achieving current densities above 1 mA cm^−2^. To further improve water splitting, g-C_3_N_4_ composites have also been investigated, offering advanced geometrical design and ease in the optimization of their sunlight trapping efficacy and recombination processes. Multielement composites have emerged as some of the most efficient materials for PEC applications, thanks to advancements in various synthesis techniques. For instance, WO_3_/BiVO_4_/Cu_2_O shows an exceptional photocurrent density of 5 mA cm^−2^ and a low R_ct_ of 61.8 Ω. However, significant engineering efforts are still required to optimize key properties, such as surface characteristics, band gap tuning, and defect control. Despite their potential, the scalability of these materials remains a major challenge due to technical constraints and high production costs. Thus, this brief review concludes that significantly more effort is required to develop efficient and scaled-up PEC technology for it to become competitive in the energy storage and conversion market.

## Figures and Tables

**Table 1:** Comparative table of the selected materials showing photoelectroactivity for the OER.

Material	Structure	Synthesis Process	Electrolyte	Photocurrent Density (mA cm^−2^)@1.23 V	Light Source	Reference
Sn-Fe_2_O_3_/CoOx-ST	Nanowires	Hydrothermal and two-step solvothermal	1 mol L^−1^ KOH	1.40	300 W Xe lamp	[105]
PdCoP-Ti:Fe_2_O_3_	Nanorods	Hydrothermal and thermal treatment	1 mol L^−1^ NaOH	2.82	300 W Xe lamp	[106]
α-Fe_2_O_3_/WO_3_	Nanorods	Hydrothermal treatment and deposition-annealing.	0.1 mol L^−1^ Na_2_SO_4_	1	300 W Xe lamp	[115]
WO_3_ quantum dots/TiO_2_	Nanowire	Hydrothermal and photoreduction	1 mol L^−1^ Na_2_SO_4_	1.5	AM 1.5 G	[116]
BiVO_4_/BiOBr	Petal-like morphology	Hydrothermal	0.5 M Na_2_SO_4_	0.2198	100 mWcm^−2^	[109]
(Ti)-doped α-Fe_2_O_3_	Nanoparticles	Hydrothermal, plasma ion implantation, and post-annealing	1 mol L^−1^ NaOH	0.55	300 W Xe lamp	[96]
WO_3_/FeOOH	Nanoplate	Hydrothermal spin-coated	0.1 M Na_2_SO_4_	2.63	300 W Xe lamp	[117]
WO_3_/BiVO_4_/Cu_2_O	Nanoworm-like, nanoparticles	Dropcastin and electrodeposition	0.5 M Na_2_SO_4_	5	xenon lamp, 100 mWcm^−2^	[118]
BiVO_4_/rGO/ Cu_2_O	Nanoparticle	Metal-organic decomposition and electrodeposition	0.5 M Na_2_SO_4_	3	300 W Xe lamp	[119]
WO_3_/FeOOH/Cu_2_O	Nanoplates	Hydrothermal, precipitation, and electrodeposition	0.2 M of Na_2_SO_4_	2.14	500 W Xe lamp	[120]
BiVO_4_/WO_3_	Nanobowl array	Lithography and two-step electro-deposition	0.2 mol L^−1^ Na_2_SO_4_	3.05	300 W Xe lamp	[121]
F:FeOOH/BiVO_4_/WO_3_	Nanoplates	Spinning calcination and and hydrothermal	phosphate buffer + 1 mol^−1^ sodium sulfite	3.1	300 W Xe lamp	[122]
CN-BiVO4/WO3	Nanoparticles	Sol-gel spin-coating	0.5 mol L^−1^ Na_2_SO_4_	0.538	150 W Xe lamp	[123]
Co-MOF/Ti-Fe_2_O_3_	Nanoparticles	Hydrothermal, calcination, and impregnation	1 mol L^−1^ NaOH	1.01	300 W Xe lamp	[104]
CoOOH/Fe_2_O_3_	Nanorods	Hydrothermal combined two-step calcination and solvothermal	1 mol L^−1^ NaOH	1.92	300 W Xe lamp	[100]
CN-FeNiOOH-CoOOH	Nanoparticle aggregates	One-step evaporation, spin-coating, photodeposition, and drop-casting.	1 mol L^−1^ KOH	3.5	300 W Xe arc lamp	[103]
α-Fe_2_O_3_/Au/TiO_2_	Nanorods	Sputtering and pulsed laser deposition	1 mol L^−1^ NaOH	1.05	100 mW cm^−2^	[97]
IACN/CoOOH/Fe_2_O_3_	Nanorods	Hydrothermal	1 mol L^−1^ KOH	2.02	300 W Xe arc lamp	[107]

## Data Availability

No new data were created.
